# Book Reviews

**Published:** 1902-11

**Authors:** 


### BOOK REVIEWS.

Psycho-Physical Exercise. Formulated by Willis B. Parks, M.D., Atlanta, Ga., 1902.

This booklet presents a system of exercise founded upon the principle of conscious co-ordination of the brain and the muscles. In order that the mind may follow the muscular movements, the movements are exceedingly slow, and the attention must be unwaveringly fixed upon them. In this way the mind becomes diverted from aberrant channels and self-control is cultivated. The exercises, ten in number, are arranged on detachable leaves, each leaf bearing the name of the physician. It is intended that the exercises shall be dispensed like an ordinary prescription. In connection with the exercises is a pamphlet containing various dietaries and selected formulæ. There is much in this system that commends itself to the reason, and the results already obtained by it show the practicality of the method.

The book can be obtained from the author.

---

A Text-Book of Pathology and Pathological Anatomy. By Dr. Hans Schmans, Bavaria, Munich, Germany. Translated from the Sixth German Edition by A. E. Mayer, M.D., New York. Edited with additions by James Ewing, M.D., New York. Illustrated with 351 engravings, including 35 colored inset plates. Lea Bros. & Co. Philadelphia and New York. 1902.

The value of Schmans's work consists in its condensation, and despite that, the inclusion of all points of the subject needed by the student. The important principles and facts of pathology are clearly set forth without unnecessary verbiage and tedious argumentative digressions. The instances and references are aptly chosen, and the book is richly and completely illustrated. The translation is well done. The book is eminently adapted to serve as a text-book.

THE PUBLIC AND THE DOCTOR. By a Regular Physician.

This book, written by Dr. B. E. Hadra, of Dallas, Texas, is designed to present to the public accurate information on medical affairs at first hand. We have nowhere seen its equal in fairness, temperateness of expression, and clearness and force in exposition. The enlightened element of the public already know dimly the facts as set forth in this volume, and are ready to agree with the views expressed. But there is also a large element, not necessarily unintelligent, but rather indifferent or inexperienced, who need to be roused or to be instructed in matters of vital importance. The book should be widely read. It would serve a noble purpose, and would go largely to the suppression of quackery and serve as a guide to the unwary.

---

QUACK MEDICINES TO BE EXCLUDED FROM THE ST. LOUIS EXPOSITION.

St. Louis is generally recognized as a city having a disproportionately large percentage of quack medicine manufacturers, and in view of the near approach of the time for holding the World's Exposition in that city, it is agreeable to learn that these unethical manufacturers will be excluded from exhibiting their wares. The following extract, taken from an exchange, relating to this matter emanates from Mr. J. A. Ockerson, chief of the Liberal Arts department of the exposition:

"Articles that are in any way dangerous or offensive; also patent medicines, nostrums and empirical preparations whose ingredients are concealed, will not be admitted to the Exposition. The director of exhibits, with the approval of the president, has the authority to order the removal of any article he may consider dangerous, detrimental to or incompatible with the object or decorum of the Exposition or the comfort and safety of the public."

The adoption of these rules indicates a growing public sentiment which will, we hope, in time even reach the medical profession and arrest the tendency too often seen in some of its members to make use of these quackish remedies.—*Exchange.*

